# Supplementary material for: Structural basis for the nuclear import and export functions of the biportin Pdr6/Kap122
Source: J Cell Biol. 2019 Apr 25;218(6):1839–52. doi: 10.1083/jcb.201812093 (PMC6548137; doi:10.1083/jcb.201812093)
Supplement: Supplemental Material (PDF) [file JCB_201812093_sm.pdf]

Supplemental material

Aksu et al., https://doi.org/10.1083/jcb.201812093

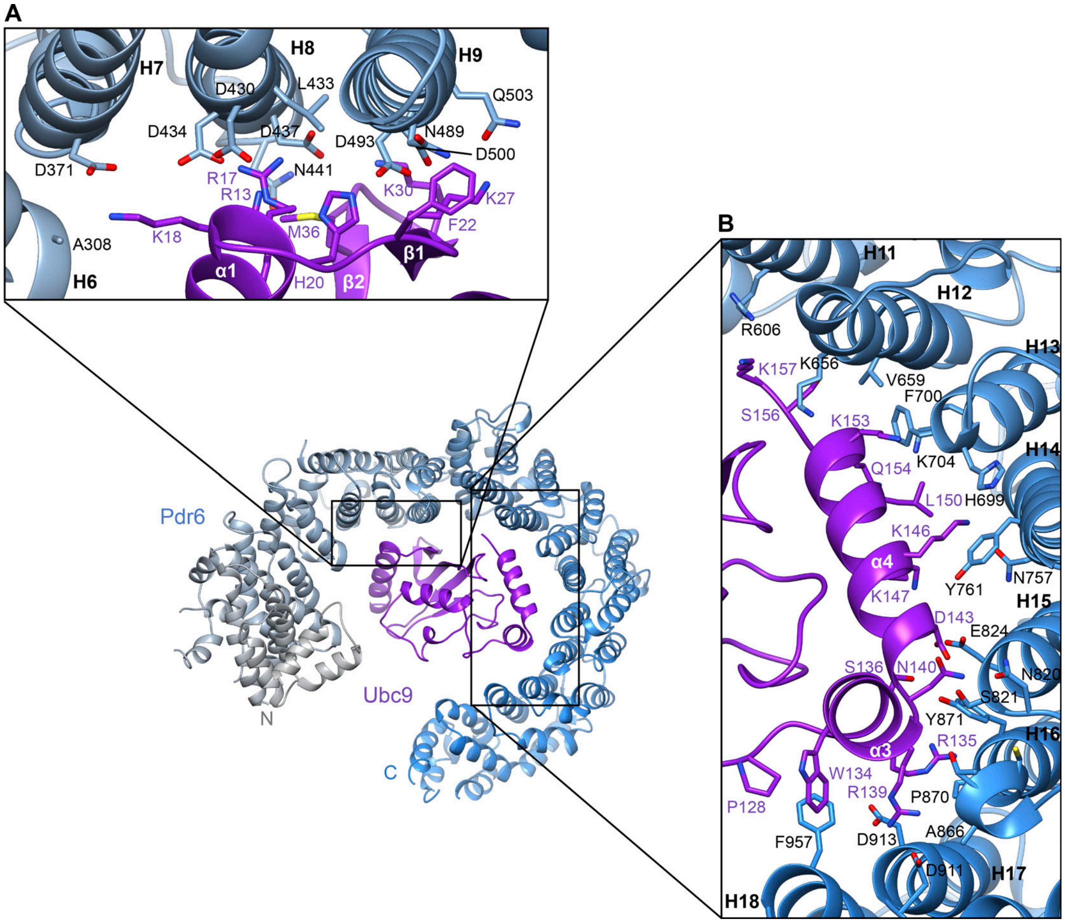

Figure S1. **Ubc9 recognition by Pdr6.** In the middle, the import complex is shown as in Fig. 2 D. Magnified views illustrate interaction interface 1 (A) and 2 (B).

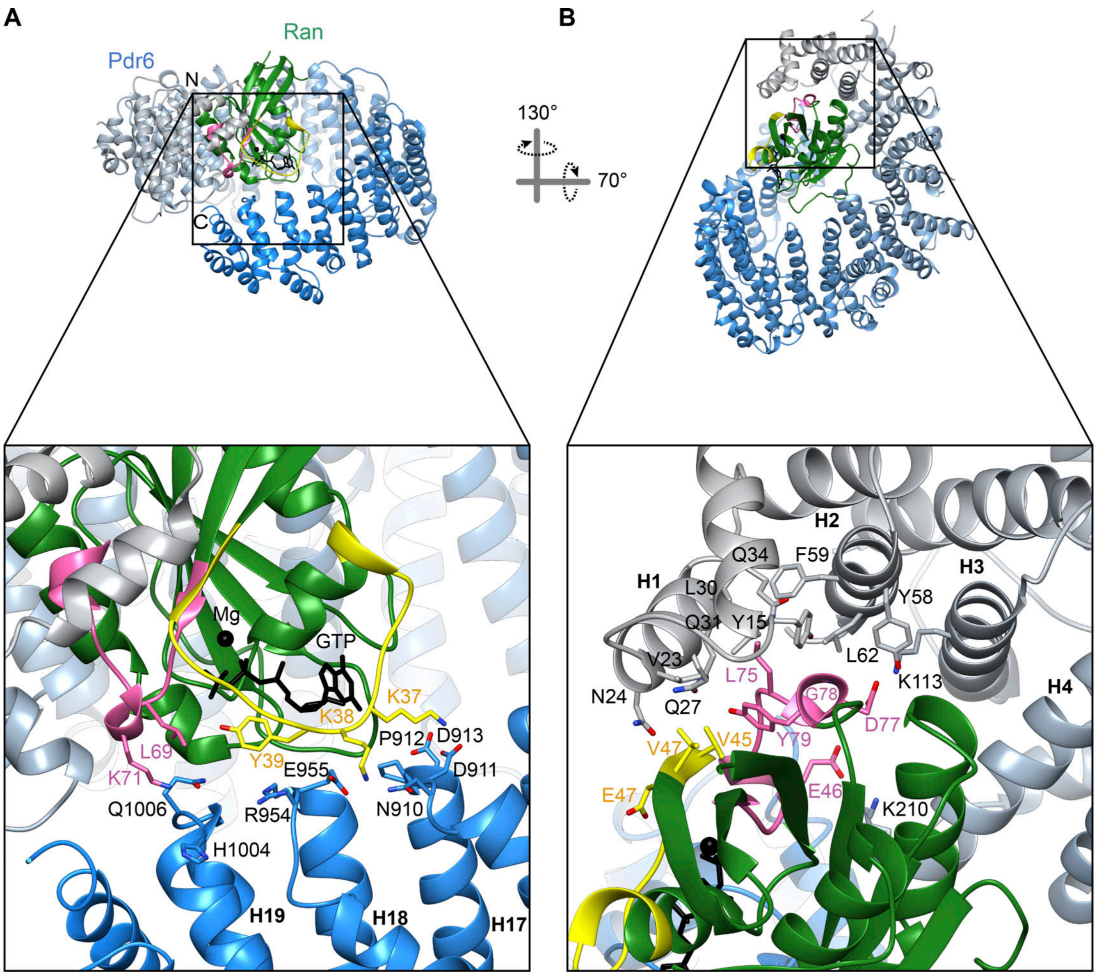

Figure S2. **Recognition of Ran's Switch I and II regions by Pdr6.** Recognition of Switch regions by C-terminal (A) and N-terminal (B) repeats of Pdr6. The color-coding of the complex is the same as in Fig. 4 B. The magnified views (bottom) show the interacting residues.

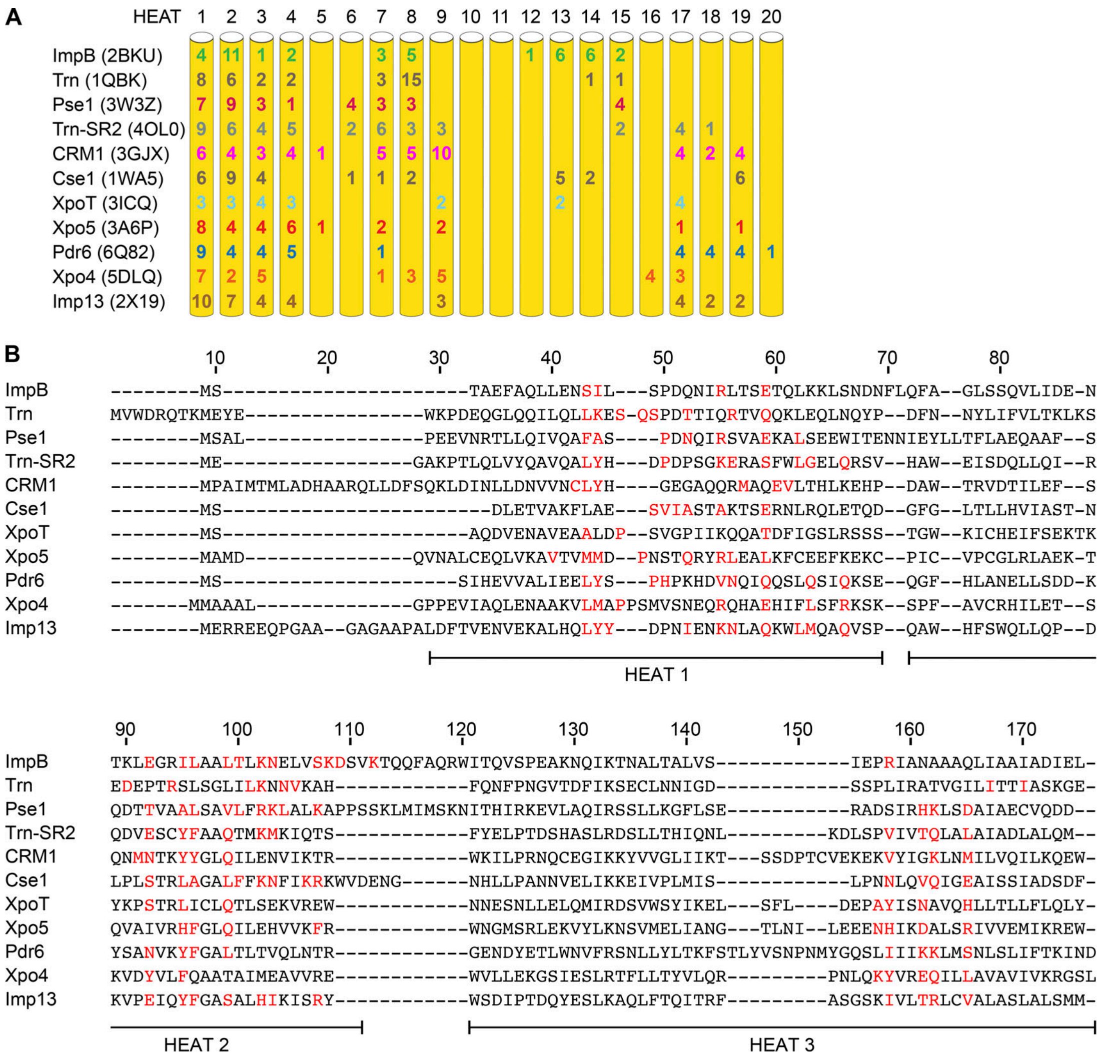

Figure S3. **NTR–RanGTP interactions. (A)** Schematic representation of RanGTP interaction interfaces of NTRs. Indicated RanGTP/Gsp1GTP•NTR structures were analyzed for NTR-RanGTP contacts, and the number of RanGTP-interacting residues in each HEAT repeat is indicated on the corresponding repeat. Note that Impβ actually lacks HEAT 20 and that Pse1 has 24 HEAT repeats, with the last four being omitted in the illustration. **(B)** Sequence alignment of the first three HEAT repeats of NTRs depicted in A. RanGTP-interacting residues are shown in red. Although the sequence conservation between these NTRs is rather low, the positional conservation of RanGTP-interacting residues is high.

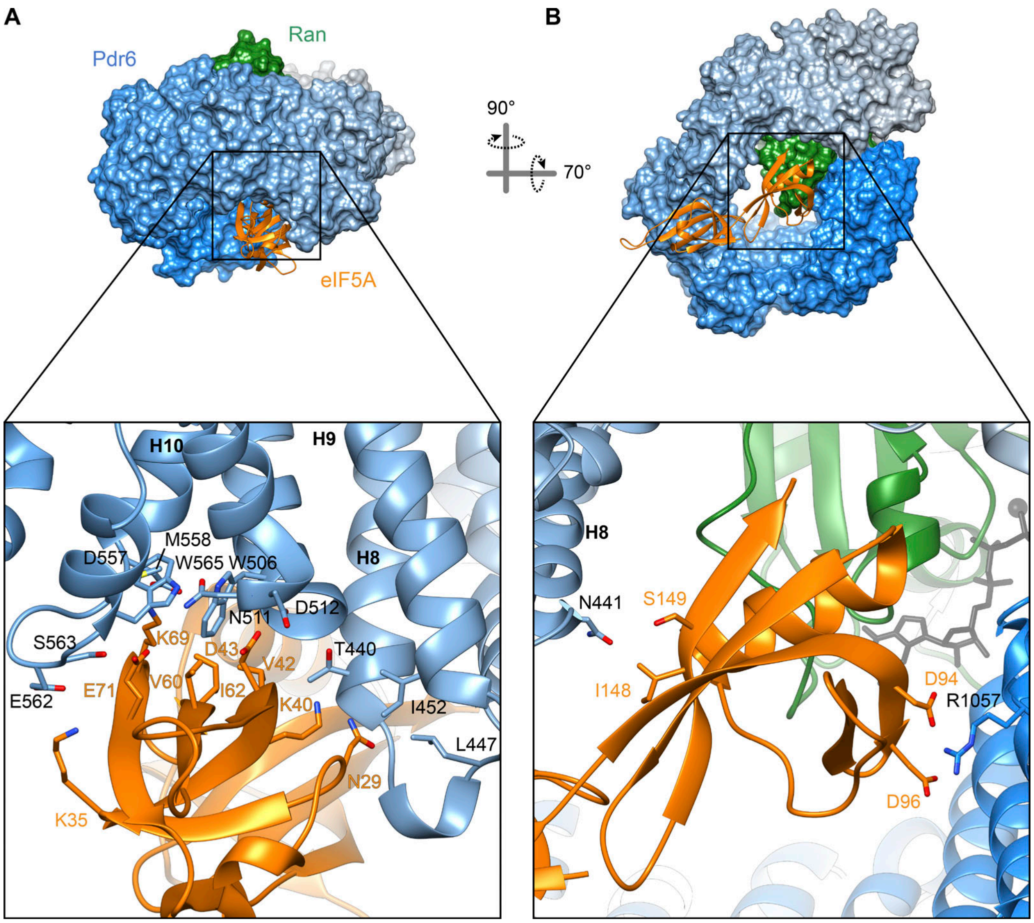

Figure S4. **Interactions of eIF5A with Pdr6.** Docking of the N-terminal (A) and C-terminal (B) domains of eIF5A on the Pdr6•RanGTP heterodimer. Pdr6 and Ran are depicted as the surface whereas eIF5A is shown as the ribbon (top). The magnified views (bottom) show the interacting residues.

Table S1. **List of plasmids used in this study**

Identifier	Construct
pMA238	H14-ZZ-bdSUMO-scPdr6
pAV366	H14-bdNEDD8-scUbc9
I-19	H10-ZZ-TEV-hsUbc9
pKG031	H14-ZZ-bdSUMO-hsRan5-180(Q69L)
pMA335	H14-ZZ-brNEDD8-sceIF5A(16-157)
pMA061	H14-ZZ-bdSUMO-hseIF5A
pMA166	H14-ZZ-bdSUMO-hseIF5A_E42R
pMA188	H14-ZZ-bdSUMO-hseIF5A_R26E
pMA189	H14-ZZ-bdSUMO-hseIF5A_K50A
pMA190	H14-ZZ-bdSUMO-hseIF5A_K50R
pMA191	H14-ZZ-bdSUMO-hseIF5A_H51A
pMA192	H14-ZZ-bdSUMO-hseIF5A_K50H51AA
pMA193	H14-ZZ-bdSUMO-hseIF5A_S75R
pMAtxn013	H14-ZZ-bdSUMO-hsImp13
pMA083	H14-bdSUMO-mmXpo4
